# Commentary: The psychological and social impact of COVID-19: New perspectives of well-being

**DOI:** 10.3389/fpsyg.2022.953147

**Published:** 2022-09-23

**Authors:** Lorena A. Flores-Plata, Anabel De la Rosa-Gómez, Dulce Díaz-Sosa, Pablo Valencia-Meléndez, Alejandrina Hernández-Posadas

**Affiliations:** Faculty of Higher Studies Iztacala, National Autonomous University of Mexico, Mexico City, Mexico

**Keywords:** telepsychology, COVID-19, well-being, psychology, Latin America

## Introduction

This paper builds on the article by Saladino et al. (2020), contributes to its goal of identifying new perspectives of intervention based on digital devices for mental health. The purpose is to broaden the discussion on the feasibility of telepsychology, its main benefits and the importance of training generations of psychotherapists in this new technological context. This will further enrich the perspective of its implementation from its massive use during the COVID-19 pandemic, for different disorders and comorbidities, specifically in Latin American countries.

## Telepsychology in the COVID-19 pandemic

The registered increase in emotional problems derived from COVID-19 has been more evident in people who already had a mental problem (with or without a diagnosis) (CDC, 2020; Rodríguez-Hernández et al., 2021; World Health Organization, 2021). The most prevalent problems during the pandemic that impacted this area of health were stress, depressive and anxious symptoms, insomnia, post-traumatic stress, suicidal ideation, domestic violence, as well as problems derived from low activity physics or teleworking (Galea et al., 2020; Mahase, 2020; Pierce et al., 2020; World Health Organization, 2020; Domínguez-Rodríguez et al., 2022; Kaluza and van Dick, 2022).

Given the mental health demands during the COVID-19 pandemic, telepsychology was a necessary element to provide services to the population (Ammar et al., 2021). Health professionals with or without training and experience, implemented online services (Tavares et al., 2020; Yuchang et al., 2022). It is known that before the pandemic only 39% of therapists used telepsychology (early adopters), and during the pandemic it increased to 85–98% (Pierce et al., 2020; Rodríguez-Ceberio et al., 2021).

Since the 1990s, the advantages and possibilities of online services have been recognized, as well as their possible limitations, ethical issues or even less positive effects, which can lead to direct effects on the patient and even on the therapist, for example when the psychotherapist in charge is not properly trained (Dworschak et al., 2022; Garcia et al., 2022) who must have a protocol of care in case of emergency, which consists of having a trusted contact or channeling institution, since the patient, in a moment of crisis where their life is in danger, could just disconnect (American Psychological Association, 2013).

## Considerations of the new era of telepsychology

Secondary effects of the pandemic (World Health Organization, 2020) included the clear increase in the use of online interventions, which might be classified as: therapist-administered treatment, treatments with a minimal assistance of the therapist and totally self-applied treatments (Glasgow and Rosen, 1982).

Telepsychology is effective in the assessment and treatment of different clinical conditions, such as depression and anxiety (Karyotaki et al., 2021; Pauley et al., 2021) substance abuse and eating disorders (Taylor et al., 2021). Also, in interventions for emotional care in COVID hospitals (Landa-Ramírez et al., 2021; Sampaio et al., 2021) and grief (Dominguez-Rodriguez et al., 2021), among others. Some meta-analyses reveal its efficacy is comparable to traditional face-to-face treatments (Cuijpers et al., 2009; Andersson et al., 2019; Sora et al., 2022).

During this pandemic period, the use of telepsychology spread throughout the world, with high-income countries being the most successful in implementing it; however, in regions with economic and technological limitations, such as Latin America, it is important to continue increasing efforts for greater reach and coverage (Argüero et al., 2021; Landa-Ramírez et al., 2021; Domínguez-Rodríguez et al., 2022). And, although there is clarity about the advantages of its use, such as reduced costs and travel time, comfort, efficiency, reduction of the stigma of going to an office and preference for the use of technology; the need to address situations of confidentiality, acceptance, closeness, and compliance with general ethical standards in telepsychology has also been highlighted (Lin et al., 2022; Sora et al., 2022; Yuchang et al., 2022). This is directly related to generating more research and training of professionals.

Based on the needs of the massive incorporation of telepsychology, the importance of developing more learning and training spaces in this area is recognized (Callan et al., 2017; Saladino et al., 2020; Baier and Danzo, 2021; Rotger and Cabré, 2022). Therapists who used this tool during the pandemic identified the need to be trained in all that telepsychology implies, to avoid falling into ethical-legal problems or to attend emergencies (e.g., in situations of suicide attempts; Perry et al., 2020; Sampaio et al., 2021), since it is generally known that there is a close relationship between the skills of the therapist, the methodology used and the remote work strategies, to predict the success of online therapy (Yuchang et al., 2022). Telepsychology training should also include stress regulation and empathy skills, as well as supervision as a measure to prevent burnout (Prime et al., 2020; Garcia et al., 2022).

## Discussion

In general, for many people, telepsychology is the key to start or continue with their psychological treatments. However, it is known that a large extent of this derived from the need for the service, without knowing or being clear about the procedures or advantages. Governmental strategies are required to allow the regulation of telepsychology as part of daily consultation activities, with supervised spaces designed by experts, under scientifically validated schemes (Pierce et al., 2020; Garcia et al., 2022). In regions such as Latin America, the incorporation of telepsychology will favor mental and physical wellbeing with access to mental health that will allow greater coverage and scope, especially if the design and development of interventions is based on the specific needs and characteristics of the patients (Domíngez-Rodríguez and De la Rosa-Gómez, 2022).

It is clear that as technology advances, health care will be directly benefited (De la Rosa-Gómez et al., 2022). However, at the same time, research, training, and dissemination of telepsychology, which represents a strategy to reach more people in an effective and efficient way, whether for emergency situations or for daily psychotherapy services, will have to be strengthened. It is important that clinics and training schools adopt telepsychology best practices guidelines and not only as a temporary solution to provide services in emergency situations. By doing so, institutions will be better positioned to meet the needs of the populations they serve (Baier and Danzo, 2021).

## Author contributions

LF-P and AD contributed to the conception of the General commentary and oriented to contribute to Telepsychology topics. DD-S emphasized ethical risk issues in the population. Together LF-P, AD, and DD-S wrote the first draft of the manuscript. Finally, PV-M and AH-P contributed to the revision of the manuscript, read, and collated citations and references. LF-P revised the submitted version. All authors contributed to the article and approved the submitted version.

## Funding

This work was supported by UNAM-PAPIIT (IT300721).

## Conflict of interest

The authors declare that the research was conducted in the absence of any commercial or financial relationships that could be construed as a potential conflict of interest.

## Publisher's note

All claims expressed in this article are solely those of the authors and do not necessarily represent those of their affiliated organizations, or those of the publisher, the editors and the reviewers. Any product that may be evaluated in this article, or claim that may be made by its manufacturer, is not guaranteed or endorsed by the publisher.

## References

[B1] American Psychological Association (2013). Guidelines for the practice of telepsychology. Am. Psychol. 68, 791–800. 10.1037/a0035001 24341643

[B2] Ammar A. Trabelsi K. Brach M. Chtourou H. Boukhris O. Masmoudi L.. (2021). Effects of home confinement on mental health and lifestyle behaviours during the COVID-19 outbreak: insights from the ECLB-COVID19 multicentre study. Biol. Sport 38, 9–21. 10.5114/biolsport.2020.96857 33795912 PMC7996377

[B3] Andersson G. Titov N. Dear B. Rozental A. Carlbring P. (2019). Internet-delivered psychological treatments: from innovation to implementation. World Psychiatry 18, 20–28. 10.1002/wps.20610 30600624 PMC6313242

[B4] Argüero A. Aguirre D. Reynoso O. Girón M. Espinosa I. Sierra M. (2021). Impacto de la telepsicología en la satisfacción de la atención a pacientes con COVID-19. Psicol. Iberoamericana 29, e293325. 10.48102/pi.v29i3.325

[B5] Baier A. Danzo S. (2021). Moving toward a new era of telepsychology in university training clinics: considerations and curricula recommendations. Train. Educ. Professional Psychol. 15, 259–266. 10.1037/tep0000359

[B6] Callan J. Maheu M. Bucky S. (2017). “Crisis in the behavioral health classroom: Enhancing knowledge, skills, and attitudes in telehealth training,” in Career Paths in Telemental Health, eds M. Maheu, K. Drude, and S. Wright (Springer International Publishing), 63–80.

[B7] CDC (2020). Coping With Stress. CDC. Available online at: https://www.cdc.gov/coronavirus/2019-ncov/daily-life-coping/managing-stress-anxiety.html

[B8] Cuijpers P. van S. Warmerdam L. Andersson G. (2009). Psychotherapy versus the combination of psychotherapy and pharmacotherapy in the treatment of depression: a meta-analysis. Depress Anxiety 26, 279–288. 10.1002/da.20519 19031487

[B9] De la Rosa-Gómez A. Flores-Plata L. A. Esquivel-Santoveña E. E. Santillán Torres Torija C. García-Flores R. Dominguez-Rodriguez A.. (2022). Efficacy of a transdiagnostic guided internet-delivered intervention for emotional, trauma and stress-related disorders in Mexican population: study protocol for a randomized controlled trial. BMC Psiquiatría 22, 537. 10.1186/s12888-022-04132-6 35941557 PMC9360670

[B10] Domíngez-Rodríguez A. De la Rosa-Gómez A. (2022). A perspective on how user-centered design could improve the impact of self-applied psychological interventions in low- or middle-income countries in Latin America. Front. Digital Health. 4, 866155. 10.3389/fdgth.2022.866155 35721795 PMC9201073

[B11] Domínguez-Rodríguez A. Herdoiza-Arroyo P. Martínez R. Bautista E. Mateu J. de la Rosa-Gómez A.. (2022). Prevalence of anxiety symptoms and associated clinical and sociodemographic factors in mexican adults seeking psychological support for grief during the COVID-19 pandemic: a cross-sectional study. Front. Psychiatry 13, 749236. 10.3389/fpsyt.2022.749236 35370841 PMC8964437

[B12] Dominguez-Rodriguez A. Martínez-Luna S. Hernández Jiménez M. De La Rosa-Gómez A. Arenas-Landgrave P. Esquivel Santoveña E. E.. (2021). A self-applied multi-component psychological online intervention based on UX, for the prevention of complicated grief disorder in the Mexican population during the COVID-19 outbreak: protocol of a randomized clinical trial. Front. Psychol. 12, 644782. 10.3389/fpsyg.2021.644782 33854466 PMC8039460

[B13] Dworschak C. Heim E. Maercker A. (2022). Efficacy of internet-based interventions for common mental disorder symptoms and psychosocial problems in older adults: a systematic review and meta-analysis. Internet Interv. 27, 100498. 10.1016/j.invent.2022.100498 35141136 PMC8810404

[B14] Galea S. Merchant R. M. Lurie N. (2020). The mental health consequences of COVID-19 and physical distancing: the need for prevention and early intervention. JAMA Intern. Med. 180, 817–818. 10.1001/jamainternmed.2020.1562 32275292

[B15] Garcia K. M. Nanavaty N. Nouzovsky A. Primm K. M. McCord C. E. Garney W. R. (2022). Rapid transition to telepsychology: health service psychology trainee responses amidst a global COVID-19 crisis from an ecological perspective. Train. Educ. Professional Psychol. 16, 87–94. 10.1037/tep0000369

[B16] Glasgow R. Rosen G. (1982). Self-help behavior therapy manuals: recent development and clinical usage. Clin. Behav. Ther. Rev. 1, 1–20.

[B17] Kaluza A. J. van Dick R. (2022). Telework at times of a pandemic: the role of voluntariness in the perception of disadvantages of telework. Curr. Psychol. 1, 1–12. 10.1007/s12144-022-03047-5 35382038 PMC8970638

[B18] Karyotaki E. Efthimiou O. Miguel C. Mass F. Furukawa T. Cuijpers P.. (2021). Internet-based cognitive behavioral therapy for depression: a systematic review and individual patient data network meta-analysis. JAMA Psychiatry 78, 361–371. 10.1001/jamapsychiatry.2020.4364 33471111 PMC8027916

[B19] Landa-Ramírez E. Sánchez-Cervantes C. T. Sánchez-Román S. Urdapilleta-Herrera E. Basulto Montero J. L. Ledesma-Torres L. (2021). Clinical psychology during the COVID-19 pandemic: experiences from six frontline hospitals in Mexico. Revista Psicoterapia. 32, 120. 10.33898/rdp.v32i120.588

[B20] Lin T. Heckman T. G. Anderson T. (2022). The efficacy of synchronous teletherapy versus in-person therapy: A meta-analysis of randomized clinical trials. Clin. Psychol. Sci. Pract. 29, 167–178. 10.1037/cps0000056

[B21] Mahase E. (2020). COVID-19: EU states report 60% rise in emergency calls about domestic violence. BMJ 369, m1872. 10.1136/bmj.m1872 32393463

[B22] Pauley D. Cuijpers P. Papola D. Miguel C. Karyotaki E. (2021). Two decades of digital interventions for anxiety disorders: a systematic review and meta-analysis of treatment effectiveness. Psychol. Med. 1–13. 10.1017/S0033291721001999 34047264 PMC9899576

[B23] Perry K. Gold S. Shearer E. M. (2020). Identifying and addressing mental health providers' perceived barriers to clinical video telehealth utilization. J. Clin. Psychol. 76, 1125–1134. 10.1002/jclp.22770 30859573

[B24] Pierce B. Perrin P. Tyler C. McKee G. Watson J. (2020). The COVID19 telepsychology revolution: a national study of pandemic-based changes in U.S. mental health care delivery. Am. Psychol. 76, 14–25. 10.1037/amp0000722 32816503

[B25] Prime H. Wade M. Browne D. (2020). Risk and resilience in family well-being during the COVID-19 pandemic. Am. Psychol. 75, 631. 10.1037/amp0000660 32437181

[B26] Rodríguez-Ceberio M. Agostinelli J. Daverio J. Benedicto G. Cocola F. Jones G.. (2021). Psicoterapia online en tiempos de COVID-19: adaptación, beneficios, dificultades. Archivos Med. 21, 548–556. 10.30554/archmed.21.2.4046.2021

[B27] Rodríguez-Hernández C. Medrano-Espinosa O. Hernández-Sánchez A. (2021). Salud mental de los mexicanos durante la pandemia de COVID-19. Gaceta Méd México. 157, 228–233. 10.24875/GMM.20000612 34667318

[B28] Rotger M. Cabré J. (2022). Therapeutic alliance in online and face-to-face psychological treatment: comparative study. JMIR Mental Health. 9, e36775. 10.2196/36775 35499910 PMC9112077

[B29] Saladino V. Algeri D. Auriemma V. (2020). The psychological and social impact of COVID-19: new perspectives of well-being. Front. Psychol. 11, 577684. 10.3389/fpsyg.2020.577684 33132986 PMC7561673

[B30] Sampaio M. Haro M. De Sousa B. Melo W. V. Hoffman H. G. (2021). Therapists make the switch to telepsychology to safely continue treating their patients during the COVID-19 pandemic. Virtual reality telepsychology may be next. Front. Virtual Reality 1, 576421. 10.3389/frvir.2020.576421 33585834 PMC7880047

[B31] Sora B. Nieto R. Montesano A. Armayones M. (2022). Usage patterns of telepsychology and face-to-face psychotherapy: clients' profiles and perceptions. Frente. Psicol. 13, 821671. 10.3389/fpsyg.2022.821671 35874378 PMC9296856

[B32] Tavares C. de Medeiros P. Silva I. de Oliveira J. Steves J. Inácio R.. (2020). The emotional impact of Coronavirus 2019-nCoV (new Coronavirus disease). Psychiatry Res. 287, 112915. 10.1016/j.psychres.2020.112915 32199182 PMC7195292

[B33] Taylor C. Graham A. Flatt R. Waldherr K. Fitzsimmons-Craft E. (2021). Current state of scientific evidence on Internet-based interventions for the treatment of depression, anxiety, eating disorders and substance abuse: an overview of systematic reviews and meta-analyses. Eur. J. Public Health. 31(31 Suppl 1), i3–i10. 10.1093/eurpub/ckab104 32918448 PMC8495688

[B34] World Health Organization (2020). Mental Health and Psychosocial Considerations During the COVID-19 Outbreak. Available online at: https://www.who.int/publications/i/item/WHO-2019-nCoV-MentalHealth-2020.1 (accessed May 15, 2018).

[B35] World Health Organization (2021). Violence Against Women. Available online at: https://www.who.int/es/news-room/fact-sheets/detail/violence-against-women (accessed May 20, 2018).

[B36] Yuchang J. Zheng Z. Peixuan Z. Junxiu A. (2022). Telepsychology: applications, advantages, and challenges. Adv. Psychol. Sci. 30, 141–156. 10.3724/SP.J.1042.2022.0014118678557

